# Interactions between Jumbo Phage SA1 and *Staphylococcus*: A Global Transcriptomic Analysis

**DOI:** 10.3390/microorganisms10081590

**Published:** 2022-08-07

**Authors:** Bingyan Zhang, Jiayi Xu, Xiaoqi He, Yigang Tong, Huiying Ren

**Affiliations:** 1 College of Veterinary Medicine, Qingdao Agricultural University, Qingdao 266109, China; 2 College of Life Science and Technology, Beijing University of Chemical Technology, Beijing 100029, China

**Keywords:** *Staphylococcus*, jumbo phage, RNA-seq, transcriptome

## Abstract

*Staphylococcus aureus* (*S. aureus*) is an important zoonotic pathogen that poses a serious health concern to humans and cattle worldwide. Although it has been proven that lytic phages may successfully kill *S. aureus*, the interaction between the host and the phage has yet to be thoroughly investigated, which will likely limit the clinical application of phage. Here, RNA sequencing (RNA-seq) was used to examine the transcriptomics of jumbo phage SA1 and *Staphylococcus* JTB1-3 during a high multiplicity of infection (MOI) and RT-qPCR was used to confirm the results. The RNA-seq analysis revealed that phage SA1 took over the transcriptional resources of the host cells and that the genes were categorized as early, middle, and late, based on the expression levels during infection. A minor portion of the resources of the host was employed to enable phage replication after infection because only 35.73% (997/2790) of the host genes were identified as differentially expressed genes (DEGs). Gene Ontology (GO) and Kyoto Encyclopedia of Genes and Genomes (KEGG) analyses showed that the phage infection mainly affected the nucleotide metabolism, protein metabolism, and energy-related metabolism of the host. Moreover, the expression of the host genes involved in anti-phage systems, virulence, and drug resistance significantly changed during infection. This research gives a fresh understanding of the relationship between jumbo phages and their Gram-positive bacteria hosts and provides a reference for studying phage treatment and antibiotics.

## 1. Introduction

*S. aureus* is a Gram-positive bacterium that has a broad distribution in nature. It is one of the most common bacteria that cause trauma infections and foodborne diseases [1,2]. An antibiotics treatment is often the choice for *S. aureus* infections. However, the long-term, high-dose, and non-standard use of antibiotics in clinics leads to drug resistance, particularly the emergence of methicillin-resistant *S. aureus* (MRSA) and vancomycin-resistant *S. aureus* (VRSA) [3,4].

Phages are a class of viruses that specifically infect bacteria, and have been widely considered as effective alternatives to antibiotics [5,6]. Compared with lysogenic (temperate) phages, lytic phages are more likely to be used as antibacterial agents. Morphologically, all known phages infecting *S. aureus* belong to the order of *Caudovirales*. These phages are categorized into three families: *Podoviridae*, *Siphoviridae*, and *Myoviridae* [7]. The genomes of *Podoviridae* and *Siphoviridae* have a genome with a modular structure. However, a few modules in the genome of *Myoviridae* are not well-separated; the order is different and certain genes are scrambled between the modules [8]. Lytic *S. aureus* phages from *Myoviridae* are likely to be the greatest candidates for phage therapy because of their wide host range [9,10].

In general, phages with a genome size less than 200 kb are classified as small-genome phages and phages with a genome size greater than 200 kb, but less than 500 kb, are classified as jumbo phages [11]. The isolation and discovery of jumbo phages have greatly enriched our understanding of biological entity diversity and evolution. The main characteristics that set jumbo phages apart from small-genome phages include their larger genome sizes, non-modular structures, dispersed genes with particular functions across the genome, the existence of RNA polymerases (RNAPs) in the phage virion that regulate gene expression, and their distance from one another [11,12].

Compared with conventional antibiotic treatments, phage therapy has several potential advantages: (1) The action mechanism of antibiotics promotes bacterial evolution and drug resistance whereas phages cause lysis and the death of bacteria when replication is complete, preventing the recurrence of bacterial infections [13]. Moreover, phages are not easily resistant to bacteria owing to DNA replication, transcription, and post-infection translation [14]. (2) The use of broad-spectrum antibiotics often disrupts the balance of intestinal flora. Phages are specialized and may kill a class of bacteria or even a single strain, making them ideal drugs for targeting and removing pathogens [15]. (3) Antibiotics must be continually given to eradicate the infection whereas phages may multiply at the area of infection and be eliminated from the body after the susceptible bacteria have been eradicated. Moreover, in vitro investigations have shown that a modest quantity of phages is sufficient to infect bacteria [16,17]. (4) Antibiotic treatments have a high risk of kidney or liver damage, but phage treatment is safe [18]. (5) Phages are more efficient than antibiotics in removing biofilms [19,20]. (6) The development of novel antibiotics has stagnated in recent years whereas a variety of phages may be readily isolated from the natural environment, allowing even drug-resistant strains to rapidly be screened [21,22]. Understanding the relationship between the phage and the host during infection is crucial to the revival of phage treatments. The present studies of the interactions between phages and hosts are mainly based on certain phages infecting Gram-negative bacteria such as *Escherichia coli* and *Pseudomonas aeruginosa* and certain phages infecting *Clostridium difficile* [23,24,25,26,27,28,29,30,31]. However, little information has been published on the effects of phage infection on the regulation of global gene expressions in *Staphylococcus* and even less is known about the potential impact on genes related to anti-phage systems, virulence, and drug resistance.

vB_StaM_SA1 (hereafter SA1), a lytic *Staphylococcus* jumbo phage, was previously isolated from the sewage of a pig farm [32]. SA1 is a phage belonging to the *Myoviridae* family that lyses a variety of *Staphylococcus*, including *S. aureus*, *S. epidermidis*, and *S. haemolyticus*, indicating that SA1 is a potential antibacterial agent against *Staphylococcus* infection. However, the interaction between phage SA1 and its host is unclear, limiting its potential clinical application.

In this study, RNA-seq was used to elucidate the interaction between phage SA1 and *Staphylococcus* during infection. The host DEGs were then enriched and analyzed and the expression of the anti-phage systems, virulence, and drug resistance genes was evaluated. Finally, three DEGs of the host and three DEGs of the phage SA1 were chosen for RT-qPCR to validate the results of RNA-seq.

## 2. Materials and Methods

### 2.1. Phage Preparation and One-Step Growth Curve

Phage SA1 was propagated in *S. lentus* JTB1-3 at 37 °C. The *S. lentus* JTB1-3 was cultured in an LB medium (5 g/L yeast extract, 10 g/L tryptone, and 10 g/L NaCl) at 37 °C. The one-step growth curve included the latent period, lysis period, and stable period, which describes the growth properties of lytic phages [33]. In short, *S. lentus* JTB1-3 was infected with phage SA1 at a MOI of about 0.1; the experiment was repeated three times.

### 2.2. Total RNA Extraction

A total of 30 mL of the *S. lentus* JTB1-3 culture at the exponential growth phase was infected with phage SA1 at a MOI of about 10; 1 mL of an uninfected JTB1-3 culture was taken out as a negative control before infection with phage SA1. The samples for RNA isolation were taken (1 mL) from the infected culture at various timepoints post-infection (5 min, 15 min, 55 min, and 125 min). The experiments were repeated three times. A FastPure Cell/Tissue Total RNA Isolation Kit V2 (Vazyme, Nanjing, China) was then used to extract the total RNA from the samples. A bioanalyzer (Agilent, Santa Clara, CA, USA) and an RNA 6000 Nano Kit (Agilent, Santa Clara, CA, USA) were used to analyze the RNA concentration and quality.

### 2.3. RNA-Seq

The Ribo-off rRNA Depletion Kit for bacteria (Vazyme, Nanjing, China) was used to deplete the rRNA before RNA-seq. For RNA-seq, certified RNA samples were sent to Majorbio (Shanghai, China). The cDNA libraries were constructed and sequenced using paired-end 300 bp reads on an Illumina HiSeq 2500 sequencer (Illumina, San Diego, CA, USA). The RNA-seq raw reads (FASTQ files) can be found in the NCBI database under accession number PRJNA836150.

### 2.4. Data Analysis

Clean reads were obtained by trimming and filtering the raw sequencing reads with SeqPrep (https://github.com/jstjohn/SeqPrep, accessed on 23 October 2020) and Sickle (https://github.com/najoshi/sickle, accessed on 23 October 2020). The clean reads were then aligned to *S. lentus* H29 (GenBank accession number NZ CP059679.1) and *Staphylococcus* phage vB_StaM_SA1 (GenBank accession number MW218148.1) utilizing Bowtie2 (http://bowtie-bio.sourceforge.net/index.php, accessed on 23 October 2020). The gene-level read counts were summarized using RSEM (http://deweylab.biostat.wisc.edu/rsem/, accessed on 23 October 2020) and the gene expression values were determined by transcripts per million reads (TPM). The DEGs defined as |log_2_FC| ≥ 1 and q < 0.05 were calculated using DESeq2 (http://bioconductor.org/packages/stats/bioc/DESeq2/, accessed on 23 October 2020). The GO and KEGG functional annotations of the DEGs were conducted using Blast2GO and Diamond, respectively. The GO and KEGG pathway enrichment analyses of the DEGs were conducted using Goatools and R, respectively, based on Fisher’s exact test [34].

### 2.5. RT-qPCR Validation of RNA-Seq

RT-qPCR was performed using Hieff^®^ qPCR SYBR Green Master Mix (Yeasen, Shanghai, China) to verify the RNA-seq data. The thermostable nuclease gene was selected as the reference gene for normalization. The primer sequences were obtained using the IDT PrimerQuest Tool (https://sg.idtdna.com/Primerquest/Home/Index, accessed on 6 May 2022) and are listed in Table S1. The changes in gene expression levels were assessed using the 2^−ΔCT^ method. Each sample was subjected to three technical replications.

## 3. Results and Analysis

### 3.1. Experimental Design of RNA-Seq after SA1 Infection

**Figure 1:**
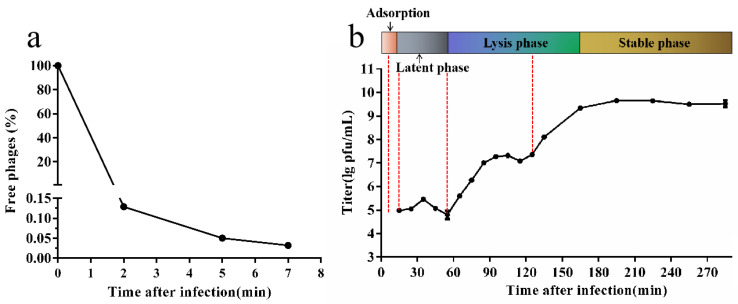
The life-cycle of phage SA1 against *S. lentus* JTB1-3. (**a**) Adsorption curve of SA1 to its host *S. lentus* JTB1-3. Free phage (%) = the number of free phages after infection/the number of free phages without infection. After incubation for 5 min, free phages accounted for only 0.05% of phages in total. (**b**) One-step growth curve of phage SA1 after incubation for 5 min. The data are expressed as means ± SD (*n* = 3).

Based on the adsorption curve (Figure 1a), the one-step growth curve was outlined by incubating the phage SA1 for 5 min (Figure 1b). According to the life-cycle of phage SA1, samples from different timepoints after infection (5 min, 15 min, 55 min, and 125 min) were chosen for RNA-seq, with uninfected bacterial cultures as the control. However, 125 min after infection, the RNA samples were discarded due to severe degradation and low RNA integrity (RIN) (Figure S1).

### 3.2. General Transcriptome Dynamics of Phage SA1 Infection

RNA-seq produced an average of 31.8 million or 30.2 million clean reads in the bacterial cultures with or without phage infection (Table 1). The clean reads were aligned to both *S. lentus* H29 and phage SA1 genomes. H29 was a suitable reference strain for JTB1-3 annotation because more than 94% of the reads mapped to the JTB1-3 genome (Table 1).

The proportion of clean reads mapping to the SA1 genome grew from 36.13% at 5 min to 66.5% at 55 min, indicating that phage SA1 took over the transcriptional resources of the host cells and the relative phage transcript increase that occurs during infection (Table 1). There was a substantial difference between the samples of the uninfected negative control group and the first timepoint according to the principal component analysis (PCA), which was used to assess the covariance between the samples (Figure S2).

### 3.3. Temporal Expression Patterns of Phage SA1

**Figure 2:**
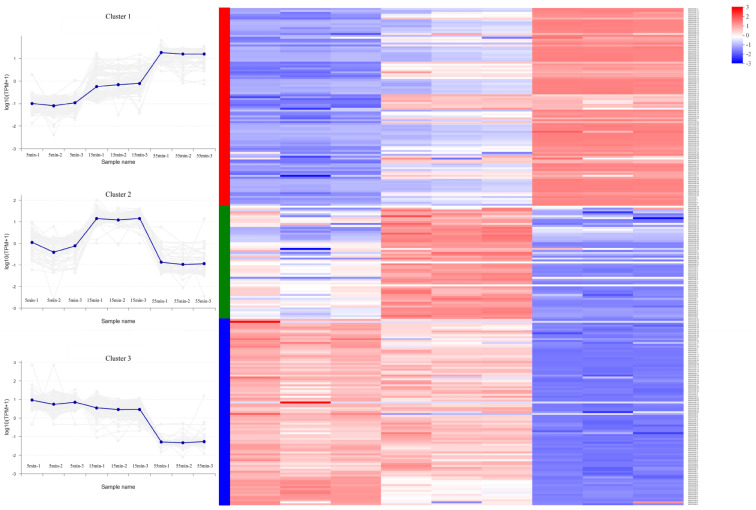
Transcriptomic profile of SA1 genes in the host *S. lentus* JTB1-3. Cluster analysis was performed using the online tool of Majorbio Cloud Platform (https://cloud.majorbio.com/page/tools/, accessed on 30 March 2022) to cluster a total of 259 genes (2 to 260) based on their TPM values and K-means method. Cluster 1: late genes. Cluster 2: middle genes. Cluster 3: early genes.

Based on the transcriptional gene levels, 259 phage genes were classified by the K-means approach into 3 clusters (Figure 2), including early genes (cluster 3), middle genes (cluster 2), and late genes (cluster 1). There was no correlation between the chromosomal location of the genes and their expression pattern. Identifying many expressed genes is challenging because most SA1 genes lack embedded systems annotations.

Most early and middle genes encoding proteins were related to DNA replication, repair, and recombination. Early genes were distinguished by their extremely high expression levels at 5 and 15 min, with no discernible variations in expression between these two periods. The functions of most early genes were also hazy. Only 7 of the 97 genes encoding proteins had a homology with known functions in the database, including glycosylase (ORF13), ATPase (ORF169), DNA gyrase B subunit (ORF18), DNA repair exonuclease SbcCD nuclease subunit (ORF256), toxic anion resistance protein (ORF51), DNA ligase (ORF63), and a DNA polymerase (ORF8).

A total of 16 genes edcoding proteins shared similarities with known functions in the database among the 59 genes that made up the middle genes. DNA replication, recombination, and repair-related proteins were encoded by genes such as DNA primase (ORF81), UvsX-like recombinase (ORF54), HNH endonuclease (ORF23), gyrase subunit A (ORF26), DNA repair exonuclease SbcCD ATPase subunit (ORF193), DNA/RNA helicase (ORF204), and DNA polymerase (ORF175 and ORF198). In particular, 4 of 7 genes annotated as RNAP belonged to the middle genes, annotated as RNAP β subunits (ORF55 and ORF77) and β’ subunits (ORF78 and ORF201), respectively. However, a gene encoding a minor virus structural protein (ORF248) was also classified as a middle gene.

Most late genes encoded phage structural proteins and host lysis-related proteins. Of the 103 late genes, 35 encoded proteins with homologs in the database. Other than the secondary virion structural proteins mentioned above, the remaining 16 genes encoding structural and assembly-related proteins were classified as late genes. Additionally, four genes encoding host lysis-related proteins, including three putative lysins (ORF211, ORF212, and ORF5) and one putative holin (ORF118), were characterized as late genes. In the late stage, ten genes encoded proteins involved in DNA replication, recombination, and repair, including four subunits of ribonucleoside diphosphate reduction (RNR) (ORF142, ORF144, ORF145, and ORF147), Holliday junction resolvase RusA (ORF117), exodeoxyribonuclease V (ORF159), DNA double-strand break repair ATPase Rad50 (ORF217), ribonuclease HI (ORF228), endonuclease fused to N-terminal Zn finger domain (ORF235), and type II DNA-binding proteins (ORF76) [35]. The enzymes encoded by these genes may be the reason for the second burst period of phage SA1. Furthermore, the late genes also included three RNAP subunits (ORF177, ORF 222, and ORF223) and a molecular chaperone GroEL (ORF 239), which may promote protein folding [36].

### 3.4. Transcriptional Response of JTB1-3 to SA1 Infection

**Figure 3:**
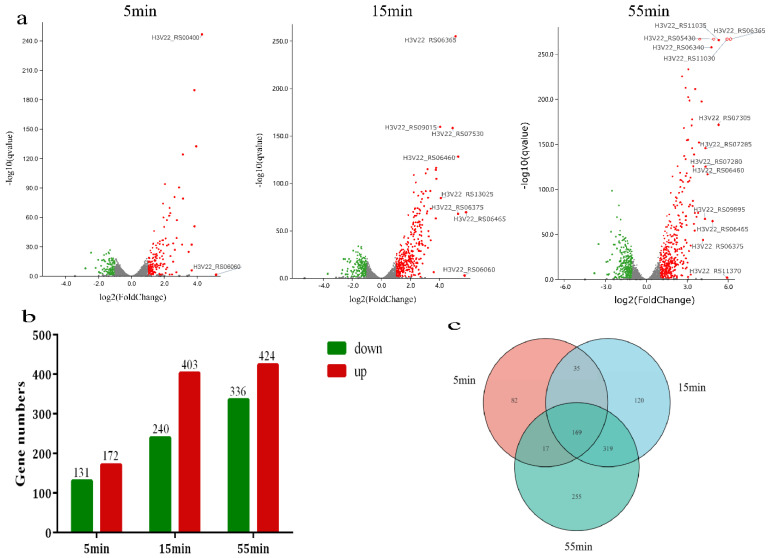
The effect of phage SA1 infection on host *S. lentus* transcription. (**a**) Volcano plots of the *S. lentus* transcriptome following phage infection compared with the uninfected control. Each dot represents an ORF, with upregulated genes shown in red and downregulated genes in green. (**b**) Number and distribution of DEGs at different infection stages. (**c**) The Venn diagram shows the intersection of the number of DEGs at each timepoint.

The DEGs of the host during infection at a MOI of about 10 were analyzed to study the responses to phage infection. According to the expression level, genes with |log_2_FC| ≥ 1 and q < 0.05 were classified as DEGs. Compared with the uninfected samples, DEG totals of 10.9% (303/2790), 23.0% (643/2790), and 27.2% (760/2790) were found at 5 min, 15 min, and 55 min after SA1 infection, respectively. The number of DEGs rose with time in response to phage infection, with more DEGs being upregulated than downregulated at all periods (Figure 3a,b). It is important to note that the sum of the DEG at each timepoint was not equal to the overall number of DEGs (997, not 1706) because specific genes were differentially expressed at several timepoints (Figure 3c). Approximately 64.27% (1796/2790) of the host *S. lentus* genes were stably expressed during phage SA1 infection (|log_2_FC|1 or q > 0.05), indicating that only a tiny portion of the bacterial resources was required for SA1 propagation.

The DEGs were classified into the eclipse phase (5 min), intracellular accumulation phase (15 min), and lysis phase (55 min) by the timepoint following phage infection. At the eclipse phase, orotate phosphoribosyltransferase (H3V22_RS00400) and a hypothetical protein (H3V22_RS06060) were the most significantly upregulated genes. It has been reported that orotate phosphoribosyltransferase is mainly involved in the pyrimidine metabolic pathway and catalyzes the transfer of the ribose phosphate group from ribose 5-phosphate 1-diphosphate to whey to form whey acid monoester (OMP) [37]. At the intracellular accumulation phase, two proteins containing surface antigen CHAP domain (H3V22_RS06365 and H3V22_RS06375), an MFS transporter (H3V22_RS06460), a protein containing a lysozyme-like domain (H3V22_RS07530), Na^+^/H^+^ antiporter family protein (H3V22_RS13025), and a winged-helix DNA-binding protein (H3V22_RS06465) were the most significant upregulated genes. The MFS transporter is considered to increase the expression of the efflux pump to extrude antibacterial molecules and make bacteria resistant to antibiotics [38,39,40,41]. Winged-helix DNA-binding protein is considered to have a role of characteristic folding and specific binding to DNA [42]. Na^+^/H^+^ antiporter family proteins are membrane proteins widely in existence and play an essential role in maintaining intracellular homeostasis [43]. At the lysis phase, the expression of a gene encoding a protein-containing CHAP domain (H3V22_RS06365) was the most significant upregulated gene; the expression level ranged from 95 before infection to 4594 at 55 min after infection (log_2_FC = 6.17). In addition to the protein highly expressed at the intracellular accumulation phase mentioned above, the LysM peptidoglycan binding domain (H3V22_RS05430), a protein related to bacterial cell wall degradation, was highly expressed in the lysis process. Moreover, LLM class flavin-dependent oxidoreductase (H3V22_RS06340) using FMN as a cofactor, two proteins involved in energy metabolism (H3V22_RS07280 and H3V22_RS07285), and one histidine ammonia-lyase (H3V22_RS09345) involved in histidine degradation were also highly expressed at this stage.

### 3.5. GO and KEGG Enrichment Analyses of Host DEGs

**Figure 4:**
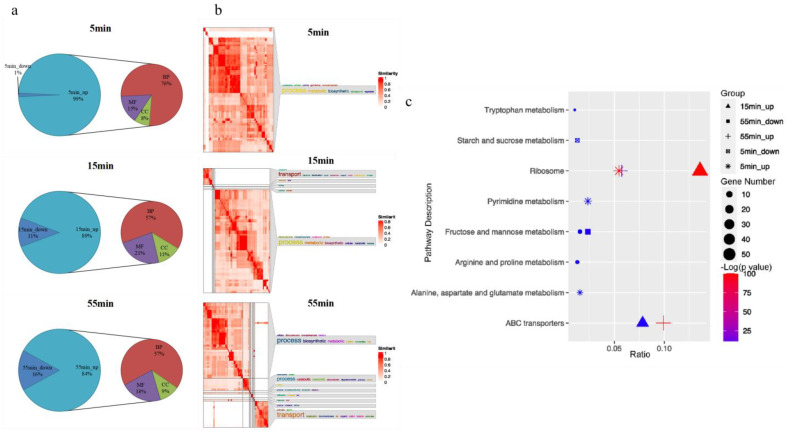
GO and KEGG analyses of host DEGs (upregulated and downregulated genes) enriched at selected points after SA1 infection. (**a**) GO analysis of the host upregulated and downregulated DEGs after phage SA1 infection. BP: biological process; CC: cellular component; MF: molecular function. (**b**) GO analysis for the biological processes of host downregulated DEGs. Terms representing each cluster are shown in a word cloud, with the size of the term representing the frequency of occurrence in the terms. (**c**) KEGG categories of host DEGs (up- and downregulated genes) enriched at selected points after SA1 infection. The shape of the point indicates the timepoints. The enrichment *p*-value of each pathway is shown as a color gradient. The size of the points represents the number of genes enriched in each pathway.

To obtain a more thorough understanding of the gene functions of the DEGs, GO and KEGG functional annotations were performed on upregulated and downregulated DEGs at each timepoint. The GO analysis showed that the DEGs had a significant functional enrichment in three stages. Although the number of upregulated and downregulated DEGs at the same time after infection did not significantly differ, the GO enrichment of the upregulated DEGs was more notable than that of the downregulated DEGs (Figure 4a). In addition, the GO enrichment suggested that the DEGs had a significant functional classification in three stages, among which biological processes (BP) were the most frequent (Figure 4a). Therefore, we used SimplifyEnrichment to visualize the upregulated DEGs involved in the biological processes at three stages after infection [44] (Figure 4b). At the eclipse phase (5 min), the upregulated DEGs were enriched to 104 BP terms, mainly related to nucleoside phosphate biosynthesis and metabolism, pyrimidine nucleoside phosphate biosynthesis and metabolism, peptide biosynthesis/metabolism, and translation. At the intracellular accumulation phase (15 min), the upregulated DEGs were enriched to 81 BP terms, mainly related to amide biosynthesis and metabolism, transmembrane transport, and protein metabolism. At the lysis process (55 min), the upregulated DEGs were enriched to 75 BP terms, mainly related to peptide biosynthesis and metabolism, amide biosynthesis/metabolism, and transport.

A total of 20.76% (207/997) of the DEGs were significantly enriched in 8 KEGG pathways (Figure 4c). After phage infection, the downregulated DEGs were significantly enriched in the starch and sucrose metabolism pathway. In contrast, the upregulated DEGs were enriched in the ribosome and ABC transporter pathways. In addition, at the eclipse phase (5 min), the upregulated DEGs were significantly enriched in the nucleic acid-related pathways (purine and pyrimidine metabolism) as well as the alanine, aspartate, and glutamate metabolism pathways.

### 3.6. Phage Infection Affects the Expression of Host Anti-Phage Systems

The host was considered to encode a CRISPR-Cas array and eight pairs of the toxin-antitoxin system (TA system). However, the genes encoding three Cas proteins (Cas2, Cas1, and Cas9) associated with CRISPR-Cas were not expressed during infection. Among the 16 TA system genes (Table 2), 56.25% were differentially expressed at one or more timepoints after infection and the number of downregulated genes was greater than that of upregulated genes. MFS transporter (H3V22_RS06460) and winged-helix DNA-binding protein (H3V22_RS06465) were upregulated at three stages after infection. In contrast, type II toxin-antitoxin system death occurrence family protein (H3V22_RS08170) and DNA-binding domain-containing protein (H3V22_RS08165) were downregulated in the early and middle stages after infection. These findings implied that the host anti-phage systems could be involved in phage infection. However, phage SA1 also inhibited the expression of several host defense genes through unidentified methods to successfully infect the host.

### 3.7. Phage Infection Affects the Expression of Host Virulence and Drug Resistance-Related Genes

To understand whether the infection of phage SA1 affected the adaptability and virulence of the host *Staphylococcus*, we analyzed the expression of the host virulence genes and drug resistance genes. We found that 40% (6/15) of the virulence genes of the host were differentially expressed at one or more timepoints (Table 3). Among them, a protein-containing hemolytic domain (H3V22_RS04090) was downregulated at three timepoints after infection [45]. Staphylococcal AgrD protein (H3V22_RS05000) and three membrane-related proteins (H3V22_RS11300, H3V22_RS13635, and H3V22_RS04680) were downregulated at 55 min after infection. SdpI/YhfL family protein (H3V22_RS07770), a multichannel integrated membrane protein that can protect toxin-producing cells from being killed, was upregulated at three timepoints after infection [46].

In addition, drug resistance-related genes were predicted and analyzed (Table 4). The results showed that 41.7% (5/12) of the drug resistance-related genes were differentially expressed at one or more timepoints after infection. LrgA superfamily protein (H3V22_RS07465), regulating extracellular cytoplasmic hydrolase activity and penicillin tolerance, was downregulated at three timepoints after infection [47]. A resistance to penicillin is produced by obtaining the *blaZ* gene [48]. Penicillin hydrolysis class A β-Lactamase (H3V22_RS08810) hydrolyzes the penicillin β-Lactam ring, which inactivates antibacterial activity; this was downregulated at three timepoints after infection [49]. In addition, three genes encoding efflux pump-related proteins were also significantly downregulated at different times after infection.

### 3.8. Verification of RNA-Seq by RT-qPCR

**Figure 5:**
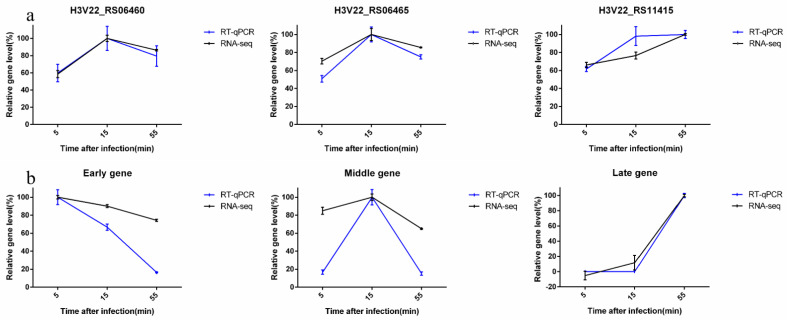
RT-qPCR verification of RNA-seq. RT-qPCR results and RNA-seq data were calculated by 2^−^^△t^ and Log_10_ (TPM) methods, respectively, and then normalized. (**a**) Verification using RT-qPCR for three TA system genes of *S. lentus* JTB1-3 upon infection. (**b**) Verification using RT-qPCR for three phage genes belonging to different stages upon infection. The data are expressed as means ± SD (*n* = 3). Graphs are plotted using GraphPad Prism 9.

Three host TA system genes differentially expressed during infection and three phage genes belonging to cluster 1, cluster 2, and cluster 3, respectively, were selected to verify the transcriptome data with RT-qPCR. It was found that three bacterial TA system genes had the same expression pattern in the two methods (Figure 5a). Although the RT-qPCR results of H3V22_RS11415 at 15 min after infection were different from the data of RNA-seq, the expression trend of the two groups of data was consistent. In addition, we randomly selected phage genes belonging to different clusters for verification. The results showed that the RT-qPCR results were compatible with the data of RNA-Seq (Figure 5b).

## 4. Discussion and Conclusions

In this work, we used RNA-seq to study the transcriptional changes in *Staphylococcus* after phage SA1 infection and highlighted the phage–host interaction. The results showed that the genes of phage SA1 were classified into early, middle, and late during infection. As an obligate parasite, the phage entirely relies on bacterial replication machinery to propagate, necessitating the genes required for DNA replication and packaging to be present at the beginning of infection, which was consistent with our findings [50,51]. 

Most early and middle genes encoded proteins involved in DNA replication, repair, and recombination. The expression of early genes was relatively high in the early and middle phases of infection, with no noticeable change. Late genes were primarily responsible for encoding structural and host lysis-related proteins, which may be crucial for the assembly, morphogenesis, and release of virus particles. In addition, there was a lack of a clear distinction between the position of SA1 genes in the genome and their expression pattern. This differed from the typical expression pattern identified in *Pseudomonas aeruginosa* phage PaP3, in which genes in each temporal category were near one another on the genome [52]. Early, middle, and late expression regions were spread across the genomes. This pattern appears to be typical of phages with large genomes such as the *Escherichia* virus T4 of 168 kbp. The function of most genes could not be speculated because they lacked a precise functional annotation.

During infection, the number of DEGs on the host increased, with upregulated DEGs always outnumbering downregulated DEGs. This discrepancy peaked at 15 min after infection when there were 67.92% more upregulated DEGs than downregulated DEGs. This result was compatible with studies on *Acinetobacter baumannii* phage φAbp and *Staphylococcus* phage SA515 [33,53]. However, it contradicted the findings on *Clostridium difficile* phage JD032 and CDHS-1 [31,54], implying that different phages have various host-regulating mechanisms. GO and KEGG enrichment analyses of the host DEGs revealed that phage infection significantly changed the nucleotide metabolism, protein metabolism, and energy metabolism of the host, which is a typical characteristic of phage infections [23,29,31,33,52,53,55,56,57,58,59].

The relationship between phages and bacteria is not only between parasite and host, but also between predator and prey [60,61]. Bacteria have developed a range of defenses against phage infection under the pressure of survival. As a result, lytic phages also have evolved to escape these antiviral defenses, including preventing adsorption, restriction modification (RM) systems, CRISPR-Cas adaptive immunity, and abortive infection (Abi) systems [62]. A bacterial toxin-antitoxin (TA) system is abundant and diversified in prokaryotes, which are a subset of Abi systems. Each pair of TA genes is composed of two genes next to one another in the genome. One gene encodes a stable toxin harmful to the host cells; the other gene encodes a homologous unstable antitoxin that can protect the host from the harmful effects of the toxin [63]. This work predicted 16 host TA system genes, of which nine were differentially expressed during SA1 infection. It indicated that the host anti-phage systems played a role in phage infection, but the phage was able to successfully “hijack” and use them for its purposes.

Bacterial antibiotic resistance is mediated by various biochemical mechanisms, the core of which is the activation of the expression of the efflux pump [38,41,64,65], which is the first defense line of *Staphylococcus* against antibiotics [66]. Efflux pumps allow the bacteria to live for an extended time, increasing the probability of spontaneous mutations that lead to high levels of resistance to specific antimicrobial agents [67,68]. Three genes encoding the efflux pump were differentially expressed, indicating that phage infection likely increased the sensitivity of the bacteria to antibiotics by downregulating the expression of efflux pump genes, providing a biological basis for phage-antibiotic synergy (PAS) [69,70,71,72,73,74].

Before RNA-seq, the one-step growth curve of phage SA1 determined the replicating process and sampling time. Unfortunately, significant RNA degradation limited the sequencing of the RNA samples from the late lysis phase. Identical degradation was also found in phages T2, T4, T7, AR9, and LUZ19 as well as in animal viruses such as herpesvirus and coronavirus [75,76,77,78,79,80]. When infecting, viruses must replicate by utilizing the molecular mechanisms of their hosts (e.g., ribosomes) to translate the messenger RNA (mRNA) of the viruses into peptides. The transfer of the gene expression from the host cell to the virus is called the host shut-off [81]. There are, reportedly, a variety of host shut-off mechanisms, but the virus-induced degradation of host mRNA has not gained sufficient attention. It is reasonable to assume that phages can efficiently perform transcription by degrading the mRNA of the host cell even though it is unclear what causes the degradation and whether the degradation has a broader influence.

In conclusion, the global transcriptional interaction between jumbo phage SA1 and its host *Staphylococcus* was described. Although SA1 is closely related to phiKZ-related jumbo phages such as AR9, we discovered that SA1 and AR9 differed in their gene expression and regulation. Less than 30% of the host genes exhibited differential expressions and the number of upregulated DEGs was greater than that of downregulated DEGs. Phage infection primarily affected the nucleotide and protein metabolism of the host and the expression of the resistance system, virulence genes, and drug resistance-related genes of the host. This study improves our understanding of how phages and bacteria interact. Future research is required to confirm several of these descriptions.

## Figures and Tables

**Table 1:** Alignment statistics of clean reads mapped to reference genome/gene sequences.

Map to Genome	Reads Number (%)			
	0 min	5 min	15 min	55 min
Total reads (million)	31.6	28.7	31.3	29.8
Map to SA1 (%)	0.01	36.13	36.86	66.51
Map to H29 (%)	94.50	59.39	60.01	30.51

**Table 2:** Transcript levels of host toxin-antitoxin (TA) system genes during phage SA1 infection ^1^.

T/A ^3^	Family	Domain	Gene	5 min ^2^		15 min ^2^		55 min ^2^	
				log_2_FC	q	log_2_FC	q	log_2_FC	q
T	-	PRK10146	H3V22_RS01825	0.000	1.000	0.000	1.000	0.000	1.000
A	-	COG4496	H3V22_RS01830	0.000	1.000	0.000	1.000	0.000	1.000
T	MazF-like	-	H3V22_RS05330	−1.045	0.000	0.488	0.037	0.296	0.125
A	MazE	-	H3V22_RS05335	0.030	0.948	−0.535	0.054	−0.406	0.240
T	-	pfam12568	H3V22_RS06460	2.374	0.000	5.307	0.000	4.484	0.000
A	-	pfam01047	H3V22_RS06465	3.483	0.000	5.840	0.000	4.846	0.000
T	-	pfam00583	H3V22_RS07715	0.567	0.000	−1.159	0.000	−1.295	0.000
A	-	COG3682	H3V22_RS07720	−0.071	0.744	0.725	0.000	0.118	0.529
T	Fic-like	COG3654	H3V22_RS08170	−1.055	0.012	−1.987	0.000	0.976	0.061
A	AbrB-like	COG2002	H3V22_RS08165	−1.643	0.000	−1.737	0.000	0.847	0.030
T	-	pfam12568	H3V22_RS08535	0.238	0.581	0.903	0.027	0.056	0.910
A	-	pfam01047	H3V22_RS08540	0.547	0.091	−1.916	0.000	0.818	0.036
T	-	pfam12568	H3V22_RS11410	0.348	0.052	0.642	0.002	0.776	0.000
A	-	COG3682	H3V22_RS11415	−0.423	0.072	1.361	0.000	3.152	0.000
T	COG2856-like	-	H3V22_RS12880	0.487	0.019	−0.274	0.320	−0.490	0.014
A	Xre-like	-	H3V22_RS12885	−0.376	0.019	−0.937	0.000	1.189	0.000

^1^ TA system was predicted by TA finder (http://202.120.12.133/TAfinder/index.php, accessed on 6 March 2022). ^2^ Positive and negative values indicate an increase and decrease in transcript levels during infection, with significant differences in red and green, respectively. ^3^ T/A: Type of protein. T: toxin; A: antitoxin.

**Table 3:** Transcript levels of host virulence genes during phage SA1 infection.

Gene ID	Description	5 min ^1^		15 min ^1^		55 min ^1^	
		Log_2_FC	q	Log_2_FC	q	Log_2_FC	q
H3V22_RS04090	Membrane protein insertion efficiency factor YidD	−1.341	0.000	−0.893	0.000	−1.153	0.000
H3V22_RS07695	NDxxF motif lipoprotein	−1.706	0.173	−2.643	0.066	−1.204	0.375
H3V22_RS09170	Capsule biosynthesis protein CapA	0.000	1.000	0.000	1.000	0.000	1.000
H3V22_RS02140	Conserved virulence factor C family protein	0.159	0.276	0.402	0.004	0.558	0.000
H3V22_RS05000	Cyclic lactone autoinducer peptide	−0.437	0.012	−0.988	0.000	−1.583	0.000
H3V22_RS09390	Disulfide bond formation protein B	−0.067	0.842	0.445	0.084	0.354	0.225
H3V22_RS00420	NFACT family protein	−0.343	0.009	0.270	0.054	0.615	0.000
H3V22_RS01190	RicA family protein	−0.299	0.037	0.426	0.003	−0.080	0.569
H3V22_RS11435	TetR/AcrR family transcriptional regulator	−0.402	0.360	0.427	0.249	0.062	0.890
H3V22_RS11300	UDP-glucose 4-epimerase GalE	0.733	0.014	−0.578	0.123	−1.204	0.015
H3V22_RS13635	UTP-glucose-1-phosphate uridylyltransferase GalU	−0.093	0.460	−0.818	0.000	−1.209	0.000
H3V22_RS04680	YihY/virulence factor BrkB family protein	−0.656	0.000	−0.914	0.000	−1.107	0.000
H3V22_RS02155	YSIRK-type signal peptide-containing protein	0.116	0.588	−0.095	0.658	−0.305	0.116
H3V22_RS07770	SdpI family protein	1.919	0.000	1.735	0.000	2.073	0.000
H3V22_RS03140	IreB family regulatory phosphoprotein	−0.013	0.957	−0.397	0.022	−0.164	0.391

^1^ Positive and negative values indicate an increase and decrease in transcript levels during infection with significant differences in red and green, respectively.

**Table 4:** Transcript levels of host drug resistance-related genes during phage SA1 infection.

Gene ID	Description	5 min ^1^		15 min ^1^		55 min ^1^	
		Log_2_FC	q	Log_2_FC	q	Log_2_FC	q
H3V22_RS07465	Antiholin-like murein hydrolase modulator LrgA	−1.459	0.000	−1.033	0.002	−1.246	0.001
H3V22_RS12740	MFS transporter	−0.008	0.972	−1.002	0.000	−1.639	0.000
H3V22_RS04235	Antibiotic biosynthesis monooxygenase	−1.118	0.000	−0.985	0.000	−1.248	0.000
H3V22_RS01910	Efflux RND transporter periplasmic adaptor subunit	−0.380	0.003	−0.780	0.000	−1.303	0.000
H3V22_RS06115	Efflux RND transporter permease subunit	−0.051	0.787	−0.095	0.577	−0.293	0.043
H3V22_RS11410	Multidrug efflux MFS transporter NorA	−0.348	0.052	−0.642	0.002	−0.776	0.000
H3V22_RS06870	SepA family multidrug efflux transporter	−0.467	0.169	−0.487	0.176	−0.275	0.477
H3V22_RS06885	TipAS antibiotic-recognition domain-containing protein	−0.039	0.867	−0.726	0.000	−0.839	0.000
H3V22_RS08810	BlaZ-like penicillin-hydrolyzing class A beta-lactamase	−1.575	0.000	−2.319	0.000	−2.334	0.000
H3V22_RS13290	Adaptor protein MecA	0.109	0.448	0.903	0.000	0.551	0.000
H3V22_RS11410	Multidrug efflux MFS transporter NorA	−0.348	0.052	−0.642	0.002	−0.776	0.000
H3V22_RS06870	SepA family multidrug efflux transporter	−0.467	0.169	−0.489	0.177	−0.275	0.477

^1^ Negative values indicate an decrease in transcript levels during infection with significant differences in green.

## Data Availability

Not applicable.

## Supplementary Materials

The following supporting information can be downloaded at https://www.mdpi.com/article/10.3390/microorganisms10081590/s1. Table S1. Primers used for RT-qPCR validation. Figure S1. The Bioanalyzer electropherograms of total RNA were isolated from the bacteria at different timepoints post-infection. After phage SA1 infection, host RNA degraded over time and the RIN (RNA integrity value) of the sample 125 min after infection was <6.5. Figure S2. The principal component analysis (PCA) graph presents the correlation between all the samples used in this study. A greater distance between points suggests a more significant difference in host *S. lentus* gene expression.

## References

[1] Teka F., Alemayehu T., Ali M.M. (2022). Methicillin-resistant *Staphylococcus aureus* antibiotic susceptibility profile and associated factors among hospitalized patients at hawassa university comprehensive specialized hospital, ethiopia. IJID Reg..

[2] Zhang Q., Xing S., Sun Q., Pei G., Cheng S., Liu Y., An X., Zhang X., Qu Y., Tong Y. (2017). Characterization and complete genome sequence analysis of a novel virulent *Siphoviridae* phage against *Staphylococcus aureus* isolated from bovine mastitis in Xinjiang, China. Virus Genes..

[3] Alonzo F., Torres V.J. (2014). The bicomponent pore-forming leucocidins of *Staphylococcus aureus*. Microbiol. Mol. Biol. Rev..

[4] Ganaie M.Y., Qureshi S., Kashoo Z., Wani S.A., Hussain M.I., Kumar R., Maqbool R., Sikander P., Banday M.S., Malla W.A. (2018). Isolation and characterization of two lytic bacteriophages against *Staphylococcus aureus* from India: Newer therapeutic agents against bovine mastitis. Vet. Res. Commun..

[5] Wills Q.F., Kerrigan C., Soothill J.S. (2005). Experimental bacteriophage protection against *Staphylococcus aureus* abscesses in a rabbit model. Antimicrob. Agents Chemother..

[6] El-Gohary F.A., Huff W.E., Huff G.R., Rath N.C., Zhou Z.Y., Donoghue A.M. (2014). Environmental augmentation with bacteriophage prevents colibacillosis in broiler chickens. Poult. Sci..

[7] Azam A.H., Tanji Y. (2019). Peculiarities of *Staphylococcus aureus* phages and their possible application in phage therapy. Appl. Microbiol. Biotechnol..

[8] Xia G., Wolz C. (2014). Phages of *Staphylococcus aureus* and their impact on host evolution. Infect. Genet. Evol..

[9] Cui Z., Song Z., Wang Y., Zeng L., Shen W., Wang Z., Li Q., He P., Qin J., Guo X. (2012). Complete genome sequence of wide-host-range *Staphylococcus aureus* phage jd007. J. Virol..

[10] Melo L.D.R., Brandão A., Akturk E., Santos S.B., Azeredo J. (2018). Characterization of a new *Staphylococcus aureus* kayvirus harboring a lysin active against biofilms. Viruses.

[11] Yuan Y., Gao M. (2017). Jumbo bacteriophages: An overview. Front. Microbiol..

[12] Nazir A., Ali A., Qing H., Tong Y. (2021). Emerging aspects of jumbo bacteriophages. Infect. Drug Resist..

[13] Kudrin P., Varik V., Oliveira S.R., Beljantseva J., Del Peso Santos T., Dzhygyr I., Rejman D., Cava F., Tenson T., Hauryliuk V. (2017). Subinhibitory concentrations of bacteriostatic antibiotics induce rela-dependent and rela-independent tolerance to β-lactams. Antimicrob. Agents Chemother..

[14] Loc-Carrillo C., Abedon S.T. (2011). Pros and cons of phage therapy. Bacteriophage.

[15] Theriot C.M., Young V.B. (2015). Interactions between the gastrointestinal microbiome and *Clostridium difficile*. Annu. Rev. Microbiol..

[16] Lee Y., Son B., Cha Y., Ryu S. (2021). Characterization and genomic analysis of PALS2, a novel *Staphylococcus* jumbo bacteriophage. Front. Microbiol..

[17] Chang Y., Shin H., Lee J.H., Park C.J., Paik S.Y., Ryu S. (2015). Isolation and genome characterization of the virulent *Staphylococcus aureus* bacteriophage SA97. Viruses.

[18] Borysowski J., Górski A. (2008). Is phage therapy acceptable in the immunocompromised host?. Int. J. Infect. Dis..

[19] Chan B.K., Turner P.E., Kim S., Mojibian H.R., Elefteriades J.A., Narayan D. (2018). Phage treatment of an aortic graft infected with *Pseudomonas aeruginosa*. Evol. Med. Public Health.

[20] Ahiwale S., Tamboli N., Thorat K., Kulkarni R., Ackermann H., Kapadnis B. (2011). In vitro management of hospital *Pseudomonas aeruginosa* biofilm using indigenous T7-like lytic phage. Curr. Microbiol..

[21] Hesse S., Rajaure M., Wall E., Johnson J., Bliskovsky V., Gottesman S., Adhya S. (2020). Phage resistance in multidrug-resistant *Klebsiella pneumoniae* ST258 evolves via diverse mutations that culminate in impaired adsorption. mBio.

[22] Sisakhtpour B., Mirzaei A., Karbasizadeh V., Hosseini N., Shabani M., Moghim S. (2022). The characteristic and potential therapeutic effect of isolated multidrug-resistant *Acinetobacter baumannii* lytic phage. Ann. Clin. Microbiol. Antimicrob..

[23] Zhao X., Shen M., Jiang X., Shen W., Zhong Q., Yang Y., Tan Y., Agnello M., He X., Hu F. (2017). Transcriptomic and metabolomics profiling of phage-host interactions between phage PaP1 and *Pseudomonas aeruginosa*. Front. Microbiol..

[24] Chueca B., Pérez-Sáez E., Pagán R., García-Gonzalo D. (2017). Global transcriptional response of *Escherichia coli* MG1655 cells exposed to the oxygenated monoterpenes citral and carvacrol. Int. J. Food Microbiol..

[25] Veses-Garcia M., Liu X., Rigden D.J., Kenny J.G., McCarthy A.J., Allison H.E., Wommack K.E. (2015). Transcriptomic analysis of Shiga-toxigenic bacteriophage carriage reveals a profound regulatory effect on acid resistance in *Escherichia coli*. Appl. Environ. Microbiol..

[26] Cech G.M., Szalewska-Pałasz A., Potrykus K., Kloska A. (2021). Virus–host interaction gets curiouser and curiouser. Part II: Functional transcriptomics of the *E. coli* DksA-deficient cell upon phage P1 vir infection. Int. J. Mol. Sci..

[27] Wicke L., Ponath F., Coppens L., Gerovac M., Lavigne R., Vogel J. (2021). Introducing differential RNA-seq mapping to track the early infection phase for *Pseudomonas phage* ɸKZ. RNA Biol..

[28] Zhong Q., Yang L., Li L., Shen W., Li Y., Xu H., Zhong Z., Chen M., Le S. (2020). Transcriptomic analysis reveals the dependency of *Pseudomonas aeruginosa* genes for double-stranded RNA bacteriophage phiyy infection cycle. iScience.

[29] Lood C., Danis-Wlodarczyk K., Blasdel B.G., Jang H.B., Vandenheuvel D., Briers Y., Noben J.P., van Noort V., Drulis-Kawa Z., Lavigne R. (2020). Integrative omics analysis of *Pseudomonas aeruginosa* virus pa5oct highlights the molecular complexity of jumbo phages. Environ. Microbiol..

[30] Sekulovic O., Fortier L.C. (2015). Global transcriptional response of *Clostridium difficile* carrying the phi CD38-2 prophage. Appl. Environ. Microbiol..

[31] Li T., Zhang Y., Dong K., Kuo C.J., Li C., Zhu Y.Q., Qin J., Li Q.T., Chang Y.F., Guo X. (2020). Isolation and characterization of the novel phage JD032 and global transcriptomic response during JD032 infection of *Clostridioides difficile* ribotype 078. mSystems.

[32] Zhang B., Sun H., Zhao F., Wang Q., Pan Q., Tong Y., Ren H. (2022). Characterization and genomic analysis of a novel jumbo bacteriophage vB_StaM_SA1 infecting *Staphylococcus aureus* with two lysins. Front. Microbiol..

[33] Yang Z., Yin S., Li G., Wang J., Huang G., Jiang B., You B., Gong Y., Zhang C., Luo X. (2019). Global transcriptomic analysis of the interactions between phage φabp1 and extensively drug-resistant *Acinetobacter baumannii*. mSystems.

[34] Klopfenstein D.V., Zhang L., Pedersen B.S., Ramírez F., Warwick Vesztrocy A., Naldi A., Mungall C.J., Yunes J.M., Botvinnik O., Weigel M. (2018). Goatools: A python library for gene ontology analyses. Sci. Rep..

[35] Nordlund P., Reichard P. (2006). Ribonucleotide reductases. Annu. Rev. Biochem..

[36] Semenyuk P.I., Moiseenko A.V., Sokolova O.S., Muronetz V.I., Kurochkina L.P. (2020). Structural and functional diversity of novel and known bacteriophage-encoded chaperonins. Int. J. Biol. Macromol..

[37] Bhatia M.B., Vinitsky A., Grubmeyer C. (1990). Kinetic mechanism of orotate phosphoribosyltransferase from *Salmonella typhimurium*. Biochemistry.

[38] Foster T.J. (2017). Antibiotic resistance in *Staphylococcus aureus*. Current status and future prospects. FEMS Microbiol. Rev..

[39] Munita J.M., Arias C.A. (2016). Mechanisms of antibiotic resistance. Microbiol. Spectr..

[40] Nawrocki K.L., Crispell E.K., McBride S.M. (2014). Antimicrobial peptide resistance mechanisms of gram-positive bacteria. Antibiotics.

[41] Peacock S.J., Paterson G.K. (2015). Mechanisms of methicillin resistance in *Staphylococcus aureus*. Annu. Rev. Biochem..

[42] Rajagopalan S., Teter S.J., Zwart P.H., Brennan R.G., Phillips K.J., Kiley P.J. (2013). Studies of iscr reveal a unique mechanism for metal-dependent regulation of DNA binding specificity. Nat. Struct. Mol. Biol..

[43] Taglicht D., Padan E., Schuldiner S. (1991). Overproduction and purification of a functional Na^+^/H^+^ antiporter coded by nhaa (ant) from *Escherichia coli*. J. Biol. Chem..

[44] Gu Z., Hübschmann D. (2021). Simplify enrichment: An R/bioconductor package for clustering and visualizing functional enrichment results. Genom. Proteom. Bioinform..

[45] Yu Z., Lavèn M., Klepsch M., de Gier J.W., Bitter W., van Ulsen P., Luirink J. (2011). Role for *Escherichia coli* YidD in membrane protein insertion. J. Bacteriol..

[46] Ellermeier C.D., Hobbs E.C., Gonzalez-Pastor J.E., Losick R. (2006). A three-protein signaling pathway governing immunity to a bacterial cannibalism toxin. Cell.

[47] Rice K.C., Nelson J.B., Patton T.G., Yang S.J., Bayles K.W. (2005). Acetic acid induces expression of the *Staphylococcus aureus* cidabc and lrgab murein hydrolase regulator operons. J. Bacteriol..

[48] Rocha G.D., Nogueira J.F., Gomes Dos Santos M.V., Boaventura J.A., Nunes Soares R.A., José de Simoni Gouveia J., Matiuzzi da Costa M., Gouveia G.V. (2022). Impact of polymorphisms in blaZ, blaR1 and blaI genes and their relationship with β-lactam resistance in *S. aureus* strains isolated from bovine mastitis. Microb. Pathog..

[49] Miragaia M. (2018). Factors contributing to the evolution of meca-mediated β-lactam resistance in *Staphylococci*: Update and new insights from whole genome sequencing (wgs). Front. Microbiol..

[50] Duffy C., Feiss M. (2002). The large subunit of bacteriophage lambda’s terminase plays a role in DNA translocation and packaging termination. J. Mol. Biol..

[51] Shen X., Li M., Zeng Y., Hu X., Tan Y., Rao X., Jin X., Li S., Zhu J., Zhang K. (2012). Functional identification of the DNA packaging terminase from *Pseudomonas aeruginosa* phage PaP3. Arch. Virol..

[52] Zhao X., Chen C., Shen W., Huang G., Le S., Lu S., Li M., Zhao Y., Wang J., Rao X. (2016). Global transcriptomic analysis of interactions between *Pseudomonas aeruginosa* and bacteriophage PaP3. Sci. Rep..

[53] Kuptsov N., Kornienko M., Bespiatykh D., Gorodnichev R., Klimina K., Veselovsky V., Shitikov E. (2022). Global transcriptomic response of *Staphylococcus aureus* to virulent bacteriophage infection. Viruses.

[54] Nale J.Y., Al-Tayawi T.S., Heaphy S., Clokie M.R.J. (2021). Impact of phage cdhs-1 on the transcription, physiology and pathogenicity of a *Clostridioides difficile* ribotype 027 strain, R20291. Viruses.

[55] Sacher J.C., Flint A., Butcher J., Blasdel B., Reynolds H.M., Lavigne R., Stintzi A., Szymanski C.M. (2018). Transcriptomic analysis of the *Campylobacter jejuni* response to T4-like phage NCTC 12673 infection. Viruses.

[56] Chevallereau A., Blasdel B.G., De Smet J., Monot M., Zimmermann M., Kogadeeva M., Sauer U., Jorth P., Whiteley M., Debarbieux L. (2016). Next-generation “-omics” approaches reveal a massive alteration of host RNA metabolism during bacteriophage infection of *Pseudomonas aeruginosa*. PLoS Genet..

[57] Baum L., Nguyen M., Jia Y., Biazik J., Thomas T. (2021). Characterization of a novel roseophage and the morphological and transcriptional response of the sponge symbiont *Ruegeria* AU67 to infection. Environ. Microbiol..

[58] Wright B.W., Logel D.Y., Mirzai M., Pascovici D., Molloy M.P., Jaschke P.R. (2021). Proteomic and transcriptomic analysis of Microviridae φx174 infection reveals broad upregulation of host *Escherichia coli* membrane damage and heat shock responses. mSystems.

[59] Mojardín L., Salas M. (2016). Global transcriptional analysis of virus-host interactions between phage ϕ29 and *Bacillus subtilis*. J. Virol..

[60] Hall A.R., Scanlan P.D., Morgan A.D., Buckling A. (2011). Host-parasite coevolutionary arms races give way to fluctuating selection. Ecol. Lett..

[61] Mirzaei M.K., Maurice C.F. (2017). Ménage à trois in the human gut: Interactions between host, bacteria and phages. Nat. Rev. Microbiol..

[62] Hampton H.G., Watson B.N.J., Fineran P.C. (2020). The arms race between bacteria and their phage foes. Nature.

[63] Van Melderen L., Saavedra De Bast M. (2009). Bacterial toxin-antitoxin systems: More than selfish entities?. PLoS Genet..

[64] Xia J., Gao J., Tang W. (2016). Nosocomial infection and its molecular mechanisms of antibiotic resistance. Biosci. Trends..

[65] Bechinger B., Gorr S.U. (2017). Antimicrobial peptides: Mechanisms of action and resistance. J. Dent. Res..

[66] Costa S.S., Viveiros M., Amaral L., Couto I. (2013). Multidrug efflux pumps in *Staphylococcus aureus*: An update. Open Microbiol. J..

[67] Piddock L.J. (2006). Clinically relevant chromosomally encoded multidrug resistance efflux pumps in bacteria. Clin. Microbiol. Rev..

[68] Ebbensgaard A.E., Løbner-Olesen A., Frimodt-Møller J. (2020). The role of efflux pumps in the transition from low-level to clinical antibiotic resistance. Antibiotics.

[69] Grygorcewicz B., Roszak M., Golec P., Śleboda-Taront D., Łubowska N., Górska M., Jursa-Kulesza J., Rakoczy R., Wojciuk B., Dołęgowska B. (2020). Antibiotics act with vB_AbaP_AGC01 phage against *Acinetobacter baumannii* in human heat-inactivated plasma blood and *Galleria mellonella* models. Int. J. Mol. Sci..

[70] Chaudhry W.N., Concepción-Acevedo J., Park T., Andleeb S., Bull J.J., Levin B.R. (2017). Synergy and order effects of antibiotics and phages in killing *Pseudomonas aeruginosa* biofilms. PLoS ONE.

[71] Torres-Barceló C., Arias-Sánchez F.I., Vasse M., Ramsayer J., Kaltz O., Hochberg M.E. (2014). A window of opportunity to control the bacterial pathogen *Pseudomonas aeruginosa* combining antibiotics and phages. PLoS ONE.

[72] Ryan E.M., Alkawareek M.Y., Donnelly R.F., Gilmore B.F. (2012). Synergistic phage-antibiotic combinations for the control of *Escherichia coli* biofilms in vitro. FEMS Immunol. Med. Microbiol..

[73] Valério N., Oliveira C., Jesus V., Branco T., Pereira C., Moreirinha C., Almeida A. (2017). Effects of single and combined use of bacteriophages and antibiotics to inactivate *Escherichia coli*. Virus Res..

[74] Rahman M., Kim S., Kim S.M., Seol S.Y., Kim J. (2011). Characterization of induced *Staphylococcus aureus* bacteriophage SAP-26 and its anti-biofilm activity with rifampicin. Biofouling.

[75] Ueno H., Yonesaki T. (2004). Phage-induced change in the stability of mRNAs. Virology.

[76] Qi D., Alawneh A.M., Yonesaki T., Otsuka Y. (2015). Rapid degradation of host mRNAs by stimulation of RNase e activity by *Srd* of bacteriophage T4. Genetics.

[77] Lavysh D., Sokolova M., Minakhin L., Yakunina M., Artamonova T., Kozyavkin S., Makarova K.S., Koonin E.V., Severinov K. (2016). The genome of AR9, a giant transducing *bacillus* phage encoding two multisubunit RNA polymerases. Virology.

[78] Mohr I. (2016). Closing in on the causes of host shutoff. Elife.

[79] Gaglia M.M., Covarrubias S., Wong W., Glaunsinger B.A. (2012). A common strategy for host RNA degradation by divergent viruses. J. Virol..

[80] Lavigne R., Lecoutere E., Wagemans J., Cenens W., Aertsen A., Schoofs L., Landuyt B., Paeshuyse J., Scheer M., Schobert M. (2013). A multifaceted study of *Pseudomonas aeruginosa* shutdown by virulent podovirus LUZ19. mBio.

[81] Sanson B., Hu R.M., Troitskaya E., Mathy N., Uzan M. (2000). Endoribonuclease RegB from bacteriophage T4 is necessary for the degradation of early but not middle or late mRNAs. J. Mol. Biol..

