# Supplementary material for: Interactions between Jumbo Phage SA1 and Staphylococcus: A Global Transcriptomic Analysis
Source: Microorganisms. 2022 Aug 7;10(8):1590. doi: 10.3390/microorganisms10081590 (PMC9414953; doi:10.3390/microorganisms10081590)
Supplement: Supplementary file 1 [file microorganisms-10-01590-s001.zip › microorganisms-1821812-supplementary.pdf]

Supplementary material

Interactions between Jumbo Phage SA1 and *Staphylococcus*: A Global Transcriptomic Analysis

Bingyan Zhang[1,2], Jiayi Xu[2], Xiaoqi He[2], Yigang Tong[2*], Huiying Ren[1*]

[1] College of Veterinary Medicine, Qingdao Agricultural University, Qingdao 266109, China

[2] College of Life Science and Technology, Beijing University of Chemical Technology, Beijing 100029, China

[*] Correspondence: renren0228@sina.com (H. R.); tong.yigang@gmail.com (Y. T.); Tel: +86-10-64451781 (Y. T.); +86-532-58957734 (H. R.)

Table S1. Primers used for RT-qPCR validation

Gene name	Forward primer	Reverse primer	Product length
H3V22_RS06460	TACTGCAACAGGGACAATAGC	ATAGCCGTCCCTAGTACCATAC	99
H3V22_RS06465	TGCAGGGAATCAACCTACAA	CTGTTAATGGCTTGAACAAGGT	120
H3V22_RS11415	AGACGTCGTCAAGATACGAAAG	GCAATTGTTTCAGCATGCTTTG	107
Late gene	TGGTTTCCAGTTTGCTTTCAG	GTCCATAAATTGAGATATTGCAACG	79
Middle gene	CAGGATGTTCAGAAGCAGATAA	ACATTCTTCCTGACATTTGGA	132
Early gene	TGTGAATGCGGAAATTGCTAAA	CCATAACGCTGGTGTCATCT	79

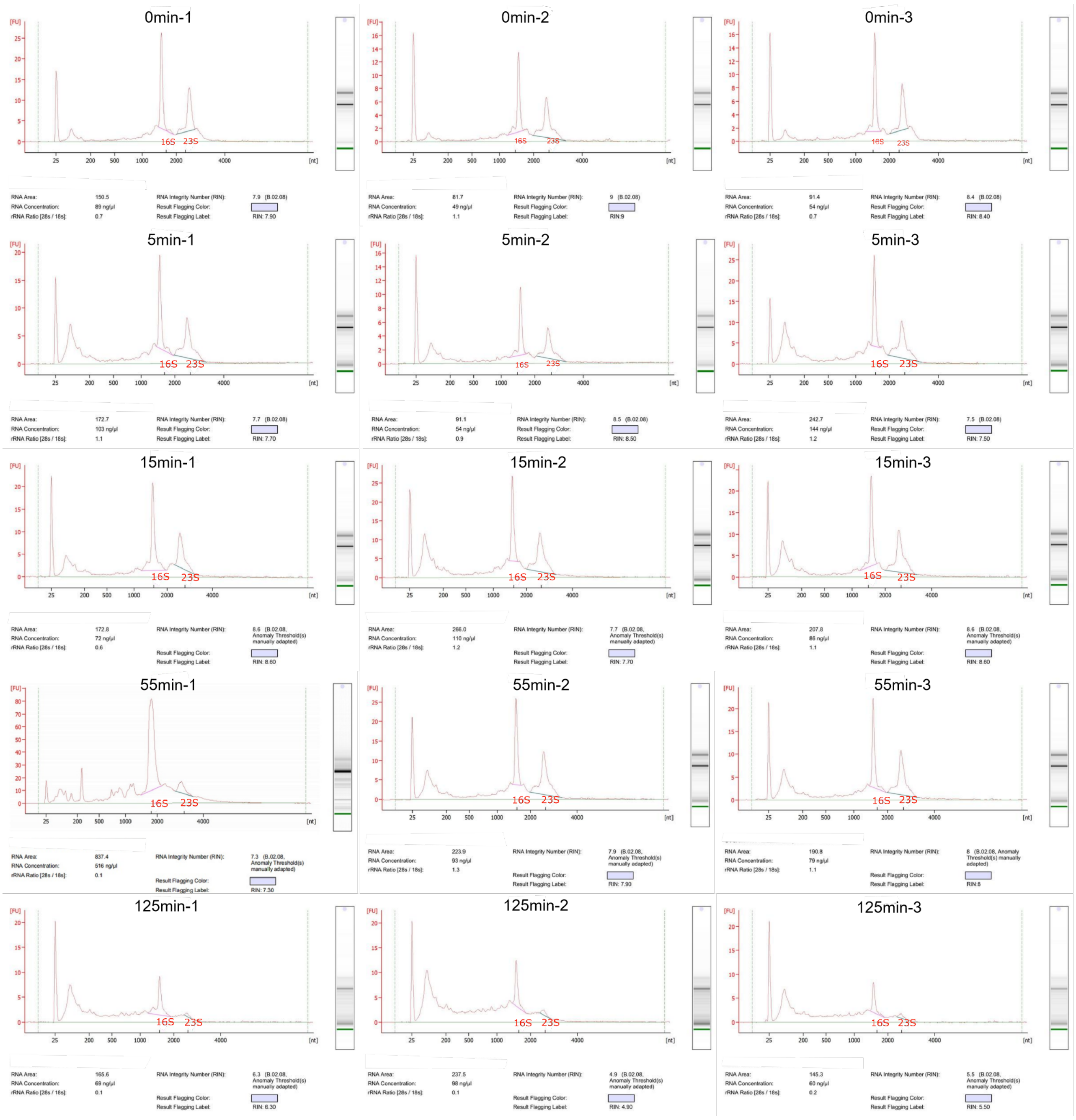

Figure S1. The Bioanalyzer electropherograms of total RNA were isolated from the bacteria at different time points post-infection. After phage SA1 infection, host RNA degraded over time, and the RIN(RNA integrity value) of the sample 125 min after infection was < 6.5.

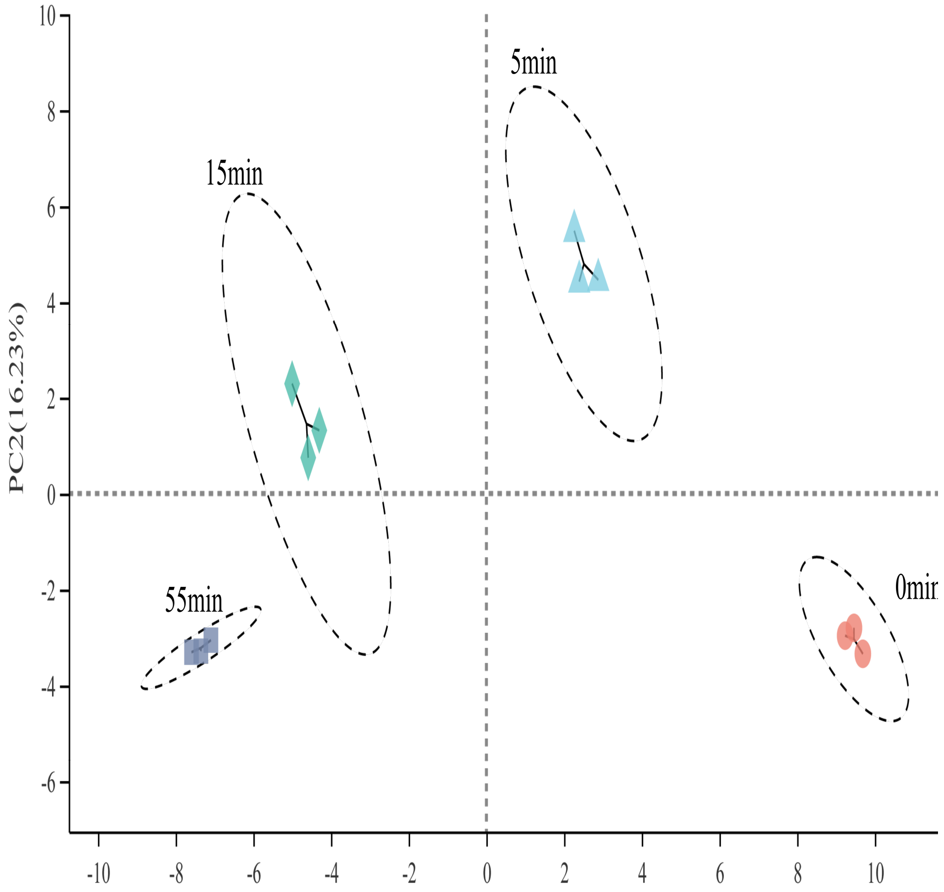

Figure S2. The principal component analysis (PCA) graph presents the correlation between all the samples used in this study. A greater distance between points suggests a more significant difference in host *S. lentus* gene expression.
