# Supplementary figures and images for: Interactions between Jumbo Phage SA1 and Staphylococcus: A Global Transcriptomic Analysis
Source: Microorganisms. 2022 Aug 7;10(8):1590. doi: 10.3390/microorganisms10081590 (PMC9414953; doi:10.3390/microorganisms10081590)

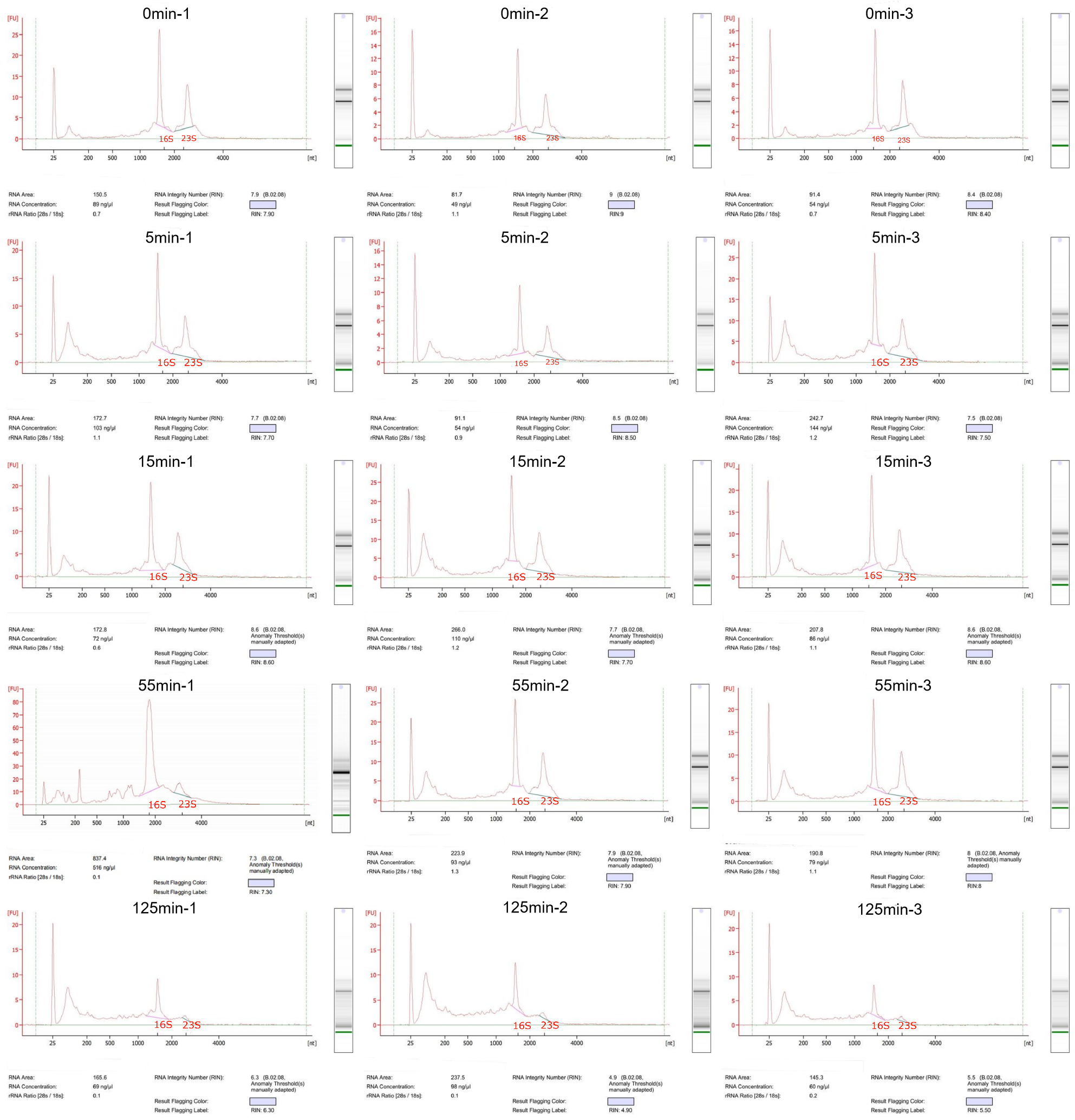

Supplement: Supplementary file 1 [file microorganisms-10-01590-s001.zip › Figure S1. RNA degradation.png]
